# The Use of Anchored Agonists of Phagocytic Receptors for Cancer Immunotherapy: B16-F10 Murine Melanoma Model

**DOI:** 10.1371/journal.pone.0085222

**Published:** 2014-01-13

**Authors:** Tereza Janotová, Marie Jalovecká, Marie Auerová, Ivana Švecová, Pavlína Bruzlová, Veronika Maierová, Zuzana Kumžáková, Štěpánka Čunátová, Zuzana Vlčková, Veronika Caisová, Petra Rozsypalová, Katarína Lukáčová, Nikol Vácová, Markéta Wachtlová, Jiří Salát, Jaroslava Lieskovská, Jan Kopecký, Jan Ženka

**Affiliations:** 1 Department of Medical Biology, Faculty of Science, University of South Bohemia, České Budějovice, Czech Republic; 2 Department of Pathology, Regional Hospital, České Budějovice, Czech Republic; 3 Department of Virology, Veterinary Research Institute, Brno, Czech Republic; Istituto Superiore di Sanità, Italy

## Abstract

The application of the phagocytic receptor agonists in cancer immunotherapy was studied. Agonists (laminarin, molecules with terminal mannose, N-Formyl-methioninyl-leucyl-phenylalanine) were firmly anchored to the tumor cell surface. When particular agonists of phagocytic receptors were used together with LPS (Toll-like receptor agonist), high synergy causing tumour shrinkage and a temporary or permanent disappearance was observed. Methods of anchoring phagocytic receptor agonists (charge interactions, anchoring based on hydrophobic chains, covalent bonds) and various regimes of phagocytic agonist/LPS mixture applications were tested to achieve maximum therapeutic effect. Combinations of mannan/LPS and f-MLF/LPS (hydrophobic anchors) in appropriate (pulse) regimes resulted in an 80% and 60% recovery for mice, respectively. We propose that substantial synergy between agonists of phagocytic and Toll-like receptors (TLR) is based on two events. The TLR ligand induces early and massive inflammatory infiltration of tumors. The effect of this cell infiltrate is directed towards tumor cells, bearing agonists of phagocytic receptors on their surface. The result of these processes was effective killing of tumor cells. This novel approach represents exploitation of innate immunity mechanisms for treating cancer.

## Introduction

According to broadly accepted cancer immunoediting hypothesis [1] cancer cells, which overcame elimination and equilibrium phases, generate the critical modifications necessary to circumvent both innate and adaptive immunological defences (escape phase). Numerous escape mechanisms include down-regulation of tumor-specific antigens [2], loss or down-regulation of MHC antigens [3], defects in antigen processing and presentation [4], expression of immune-inhibitory ligands on tumor cells [5], induction of central or peripheral tolerance [6] or generation of an immunosuppressive tumor microenvironment [7].

While the most important component of anti-tumor immunity is represented by cytotoxic T lymphocytes [8], among cells of innate immunity, NK cells seem to play the most significant role [9]. The role of other innate immunity cells is much less explored and almost nothing is known about recognition of tumor cells by unarmed macrophages or granulocytes [10].

Nevertheless, Cui et al. [11] and Hicks et al. [12] showed that mice with a SR/CR mutation, enabling recognition of tumor cells via a so far unknown mechanism, successfully killed tumor cells. *In vitro* experiments demonstrated that cells of innate immunity (NK cells, macrophages, neutrophils) were responsible for cancer cell killing. Exploitation of pattern recognition receptor (PRR) agonists to stimulate innate signalling pathways [13] is another partially successful approach to treatment of cancer. Complex mechanism of PRR agonist action consists in the production of interferon type I and other proinflammatory cytokines, enhanced maturation of dendritic cells, secretion of Th1 cytokines, antigen cross-presentation, activation of NK cells and suppression of regulatory T cells and tumor associated macrophages [14]. Clinical trials focused on usage of synthetic ligands of the Toll-like receptors (TLR) 3,7,9 for tumor treatment [15].

However, besides the fact that activation of signalling receptors (mainly TLR) leads to establishment of strong answer at the level of innate immunity, tumor infiltrating immune cells must recognize tumor cells as the true targets of their attack. We suggest manipulating phagocytic cells (an important component of inflammatory infiltrate) to be able to find their targets by coupling agonists of phagocytic receptors on the surface of tumor cells to obtain a strong antitumor effect. This effect can be dramatically enhanced by simultaneous treatment of TLR receptors with an agonist (e.g., LPS).

## Materials and Methods

### Ethics Statement

All of the experimental procedures were conducted in accordance with the law of the Czech Republic on the use of experimental animals, safety and use of pathogenic agents. The study was approved by the Institute of Parasitology, Biology Centre of the Academy of Sciences of the Czech Republic and Institutional and National Committees (protocols no. 138/2008).

Anaesthesia of mice (used during transplantation of melanoma cells) was based on intraperitoneal injection of Ketamine.HCl (75 mg/kg) and Xylazine.HCl (75 mg/kg). For survival analysis mice were monitored twice a day. Where tumor growth restricted an animal's ability to move normally or to eat or drink then mice were sacrificed via cervical dislocation.

### Chemicals

Tissue culture media and supplements, laminarin from *Laminaria digitata*, mannan from *Saccharomyces cerevisiae,* lipopolysaccharides (LPS) from *Escherichia coli*, lipoteichoic acid (LTA) from *Bacillus subtilis*, dithiothreitol (DTT), Tris(2-carboxyethyl)phosphine hydrochloride (TCEP), DAPI, and f-MLF (N-Formyl-methioninyl-leucyl-phenylalanine) were obtained from Sigma-Aldrich (St. Louis, MO, USA). 4-(*N*-Maleimidomethyl) cyclo-hexanecarboxylic-acid N-hydroxysuccinimide ester (SMCC) was purchased from Thermo Scientific (Erembodegem, Belgium). Biocompatible Anchor for cell Membrane (BAM, Mw 4000) and N-(Succinimidyloxy-glutaryl)-L-α-phosphatidylethanolamine, Dioleoyl (DOPE) were obtained from NOF EUROPE (Grobbendonk, Belgium). Anti-CD11b-FITC conjugate was obtained from MACS Miltenyi Biotec.

Monomannosyldekalysine was synthesized by Vidia (Prague, Czech Republic). Mannose-(G)_5_-(K)_12_, mannose-(G)_5_-(K)_10_-STE (STE means stearic acid), f-MLF-(G)_5_-(K)_12_, f-MLF-(G)_5_-(K)_10_-STE, MLF-(G)_5_-(K)_10_-STE, and f-MLFKK were synthesized by Schafer-N (Copenhagen, Denmark).

### Synthesis of laminarin-BAM, mannan-BAM, f-MLFKK-BAM, and f-MLFKK-DOPE

First, both aminated laminarin and mannan were prepared by reductive amination [16]. Laminarin (mannan) solution in an environment of ammonium acetate was reduced by natrium cyanoborohydride at pH 7.5 and 50°C for five days. Solution was further dialyzed using MWCO 3500 dialysis tubing (Serva, Heidelberg, Germany) against PBS at 4°C overnight. Peptide f-MLFKK already contained an amino group.

Binding of BAM (contains one aliphatic chain) or DOPE (two aliphatic chains) on amino group of laminarin (mannan, f-MLFKK) was performed at pH 7.3 according to Kato et al. [17]. During one hour at room temperature N-hydroxysuccinimide (NHS) group of BAM resp. DOPE reacted with amino group of laminarin (mannan), or with ε-amino group of lysine respectively. Solutions obtained (in PBS) were stored frozen at –20°C until use.

### Synthesis of laminarin-SMCC, mannan-SMCC, f-MLFKK-SMCC, and their *in vivo* and *in vitro* application

According to manufactureŕs instructions (Thermo Scientific, Pierce Protein Biology Products), similarly to the previous paragraph, NHS group of SMCC reacted with amino group of aminated laminarin and mannan, or with ε-amino group of lysine in f-MLFKK (equimolar amounts) respectively. To guarantee binding of SMCC containing ligands to tumor cells, it was necessary to ensure existence of –SH groups on the cells. It was accomplished according to Christiaansen et al. [18] by reduction of cystines. In our *in vivo* experiments we used 50 mM solution of TCEP in PBS for this purpose. This solution was injected intratumorally (i.t.) one hour before application of laminarin-SMCC, mannan-SMCC or f-MLFKK-SMCC solutions (in PBS). In our *in vitro* experiments we used 5 mM solution of TCEP in PBS and one hour incubation on ice.

### Cell lines and mice

Murine melanoma B16-F10 cells and peritoneal macrophages PMJ2R were purchased from American Type Culture Collection (ATCC, Manassas, VA). Both cell lines were cultivated in RPMI 1640 (Sigma-Aldrich, USA) supplemented with 10% foetal calf serum (FCS, PAA, Austria) and antibiotics. Cells were maintained at 37°C in humidified air with 5% carbon dioxide.

Female SPF C57BL/6 mice were obtained from Charles River Laboratories (Sulzfeld, Germany). Mice were housed in plastic cages with wood-chip bedding situated in a specific-pathogen free room with a constant temperature of 22°C and a relative humidity of 65%. Pellet diet and water were sterilized. Mice were housed in a 12/12-hour photoperiod environment with free access to food and water. Mice weighing 18–20 g were used in experiments.

### Tumor transplantation

4×10^5^ B16-F10 cells per mouse in 0.1 ml RPMI without FCS were inoculated subcutaneously (s.c.) in a shaved area on the right flank.

### Treatment and evaluation of treatment

Mice were randomised in groups twelve days after tumor transplantation. Therapies started immediately (intratumoral applications of 50 μl of corresponding solutions). Since this time, mice were kept individually.

Tumors were measured every second day using callipers. Volume was calculated as previously described [19] using formula V = π/6 AB^2^ (A denotes the largest dimension of tumor mass and B denotes the smallest dimension).

### Mean reduction of tumor growth (%)

Reduction of tumor growth (compared with control) was determined as follows:



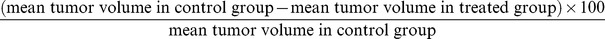



Mean (in %) of values measured on days 4, 6, 8, 10, 12 and 14 after beginning of therapy was calculated and marked as “mean reduction of tumor growth”.

### Analysis of cell infiltrate using flow cytometry. Cytokine assay

The tumor was excised from the mouse which had been euthanized via cervical dislocation. It was then gently washed with cold RPMI 1640, cut into small pieces and placed into 1 ml cold RPMI 1640 containing 0.33 mg/ml Liberase DL and 0.2 mg/ml DNase I (both Roche Diagnostics, Germany). After 1 h incubation on a rotary shaker at 37°C, clumps of tissue aggregates were centrifuged at 160 *g* for 10 min at 4°C. Supernatant was used for IL-1 beta, TNF alpha, IL-6, (ELISA, eBioscience), and IL-8 (R&D Systems) determination performed according to manufacturer recommendations. The resulting pellet was gently passed through a plastic strainer (70 µm, BD Biosciences, USA) into cold PBS (pH 7.3) and washed by centrifugation at 160 *g* for 10 min at 4°C. Cells were then transferred into 96-well plate (Corning Incorporated, USA) and analyzed using flow cytometry.

Cells were incubated with a solution of pre-diluted specific monoclonal antibodies recognizing mouse surface antigens (all eBioscience, USA) in PBS for 20 min at 4°C. In the cell suspension obtained from the tumor, the following leukocyte subtypes were determined: leukocytes (anti-Mouse CD45 PerCP-Cy5.5; clone 30-F11; 0.2 mg/ml), B cells (anti-Mouse CD19 APC; clone eBio1D3; 0.2 mg/ml), T cells (anti-Mouse CD3e FITC; clone 145-2C11; 0.5 mg/ml), CD4+ T cells (anti-Mouse CD4 APC; clone GK1.5; 0.2 mg/ml), CD8+ T cells (anti-Mouse CD8a; clone 53-6.7; 0.2 mg/ml), NK cells (anti-Mouse NK1.1 PE; clone PK136; 0.2 mg/ml), granulocytes (anti-Mouse Ly-6G (Gr-1) Alexa Fluor 700; clone RB6-8C5; 0.2 mg/ml) and monocytes/macrophages (MF cells) (anti-Mouse F4/80 Antigen PE-Cy7; clone BM8; 0.2 mg/ml). Labelled cell samples were washed twice in PBS by centrifugation at 160 *g* for 2 min at 4°C and analyzed using a BD FACSCanto II flow cytometer (BD Biosciences, USA), equipped with two lasers with excitation capabilities at 488 nm and 633 nm. Twenty thousand events were measured in each suspension in three independent repetitions. The labelled cell populations were analysed using BD FACSDiva software 6.1.3. Absolute numbers of leukocyte subsets were quantified using CountBright^TM^ absolute counting beads (Invitrogen, USA). The control of all specific monoclonal antibodies recognizing mouse surface antigens was performed on a sample of splenocytes in each interval of the experiment. Cell count was recalculated and expressed as cells/mm^3^ of tumor tissue.

### Histology

Tumors were fixed with 4% neutral solution of formaldehyde. Paraffin blocks were prepared. Sections were stained by hematoxylin/eosin.

### Lung metastases

Lungs fixed with 4% neutral solution of formaldehyde were examined with the aid of a dissecting microscope. The presence of metastases (black points) was evaluated.

### *In vitro* analysis of the cytotoxic effect of macrophages activated by a TLR ligand on melanoma cells bearing phagocytic receptors

The assay was based on the principle described previously [20]. Murine B16-F10 melanoma cells grown to confluency in 96 well tissue culture plate (Nunc, Roskilde, Denmark) were incubated (30 min, 37°C) with a solution of phagocytic receptor agonists (0.02 mM laminarin–BAM or 0.02 mM mannan-BAM or 0.05 mM f-MLFKK-BAM in culture medium) and subsequently washed. Cells of murine macrophage cell line PMJ2R were preincubated with LPS (1 μg/ml) for 2 hours at 37°C, then they were washed, resuspended in RPMI 1640, 10% FCS and added to B16-F10 in the ratio 5∶1. This mixture was incubated for 4 hours at 37°C. After incubation, PMJ2R and dead cells were carefully washed off. Living B16-F10 melanoma cells were released by trypsinisation. Trypan blue excluding cells were quantified with a haemocytometer.

### Cell signalling

2×10^5^ of each, B16-F10 and PMJ2R cells were seeded together in the presence of laminarin-BAM, or B16-F10 cells with covalently bound laminarin-SMCC were used. After indicated time of incubation the cells were lysed in a modified RIPA buffer (1% Nonidet P-40, 0.25% sodium deoxycholate, 1 mM EGTA, 150 mM NaCl, and 50 mM Tris-HCl (pH 7.5)) in the presence of protease inhibitors (10 μg/ml aprotinin, 1 μg/ml leupeptin, 1mM phenylmethylsulfonyl fluoride, 1 μg/ml pepstatin) and phosphatase inhibitors (25 mM sodium fluoride and 2 mM sodium orthovanadate). The cell lysates were mixed with 4x Laemmli sample buffer, than proteins were separated by SDS-PAGE, and transferred to Immobilon-P membrane. The blots were incubated with anti-phospho-NF-κB p65 (Ser536, Cell Signalling) and with anti-β-actin Santa Cruz Biotechnology) antibody at dilution 1∶1000. Proteins were visualized by ECL (enhanced chemiluminiscence, Pierce), and their abundance was determined using CCD image system (ChemiDoc^TM^ MP Imaging System, BIO-RAD) and ImageLab software.

### Capability of BAM and DOPE to anchor molecules to cell membranes

Conjugation of BAM or DOPE with B-Phycoerythrin (PE) was performed at pH 7.3 in the dark as previously described [17]. One hour lasting interactions of PE-BAM, PE-DOPE and PE with 1×10^5^ melanoma cells were performed at 37°C in the dark in triplicates. After centrifugation (2 min. 4°C, 400 g) supernatants were harvested and its fluorescence measured by Infinite M200 reader (Tecan, Switzerland) at 545 nm.

### Statistical analysis

Statistical analysis was performed using two-tailed Student's t-test. Mouse survival was evaluated using Kaplan-Meier test (MedCalc).

## Results

### The effect of laminarin in cancer therapy

#### The effect of anchored laminarin (laminarin-BAM) on tumor growth and its synergy with LPS

**Figure 1:**
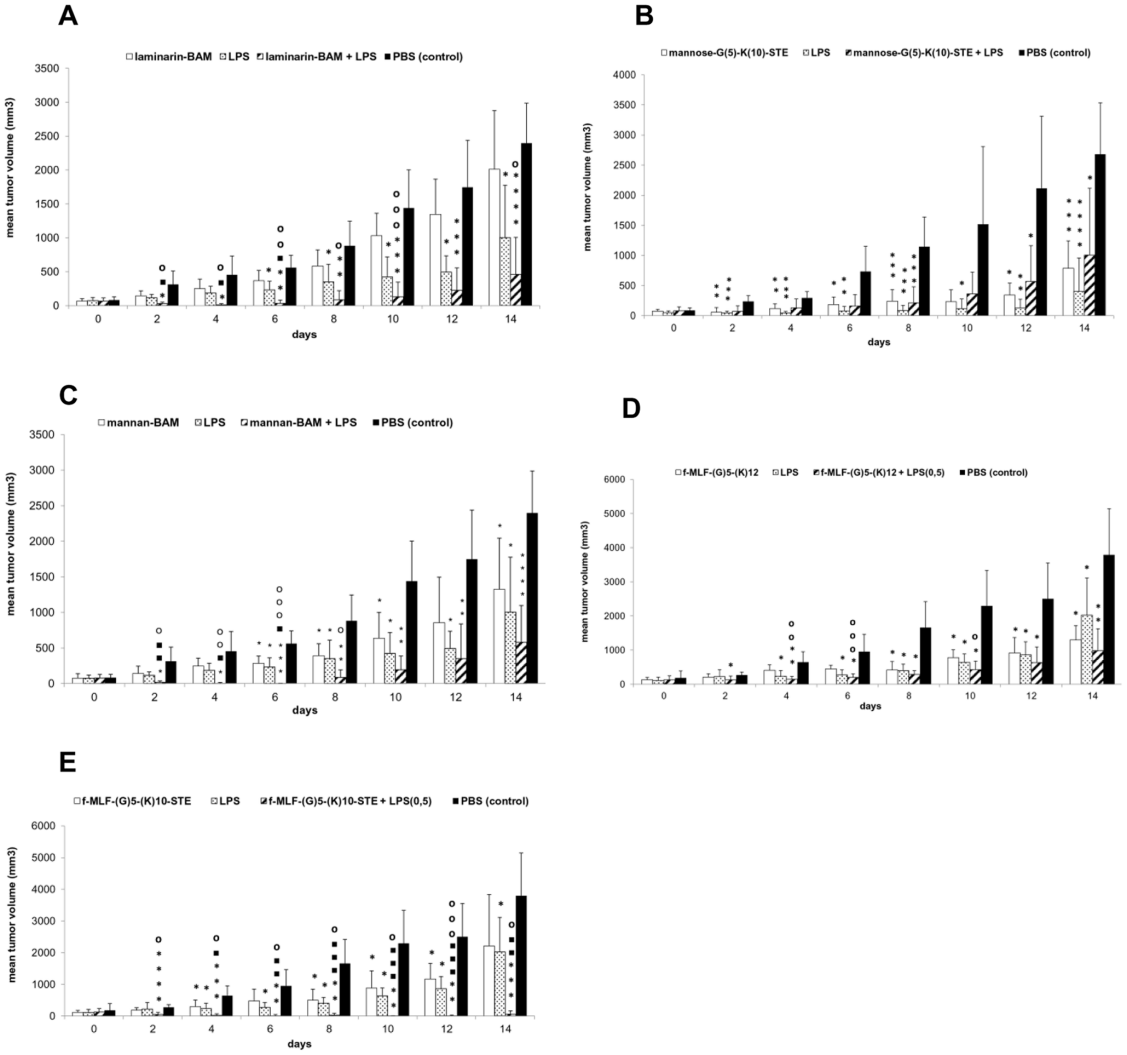
The effect of anchored ligands of phagocytic receptors on tumor growth and their synergy with LPS. C57BL/6 mice (females) were inoculated with 4×10^5^ murine melanoma B16-F10 cells per mouse in 0.1 ml RPMI subcutaneously in a shaved area on the right flank. Mice were randomized in groups of 5–6 twelve days after tumor transplantation. Therapies started immediately by intratumoral applications of 50 μl of corresponding solutions and continued every second day for 10 days (together 6 doses). After therapy had commenced, mice were kept individually. Tumors were measured every second day for 14 days and their volume was calculated. (A) Anchored laminarin (laminarin-BAM). Groups of 5 mice obtained 0.2 mM laminarin-BAM in PBS, LPS (0.5 mg/ml PBS), mixture of 0.2 mM laminarin-BAM and LPS (0.5 mg/ml) in PBS, and PBS alone. (B) Anchored mannose. Groups of 6 mice obtained 3 mM mannose-(G)_5)_-(K)_10_-STE in PBS, LPS (0.5 mg/ml PBS), mixture of 3 mM mannose-(G)_5_-(K)_10_-STE and LPS (0.5 mg/ml) in PBS, and PBS alone. (C) Anchored mannan. Groups of 5 mice obtained 0.2 mM mannan-BAM in PBS, LPS (0.5 mg/ml PBS), mixture of 0.2 mM mannan-BAM and LPS (0.5 mg/ml) in PBS, and PBS alone. (D) Anchored formylpeptide receptor agonist by oligolysin. Groups of 6 mice were injected with 3 mM f-MLF-(G)_5_-(K)_12_ in PBS, LPS (0.5 mg/ml PBS), mixture of 3 mM f-MLF-(G)_5_-(K)_12_ and LPS (0.5 mg/ml) in PBS, and PBS alone. (E) Anchored formylpeptide receptor agonist by stearic acid. The same regime as in (D), 3 mM f-MLF-(G)_5_-(K)_10_-STE used instead of 3 mM f-MLF-(G)_5_-(K)_12._ *P≤0.05, **P≤0.01, ***P≤0.005, ****P≤0.001 compared to control ■P≤0.05, ■■P≤0.01, ■■■P≤0.005 compared to LPS **o**P≤0.05, **oo**P≤0.01, **ooo**P≤0.005 compared to the ligand.

Melanoma B16-F10 was transplanted into 20 C57BL/6 mice. Twelve days after this, mice were randomised in four groups containing five mice each. On this day, tumor volume was measured and tumor therapy started immediately thereafter. As Figure 1A shows, laminarin-BAM did not have a significant effect on tumor growth. The effect of LPS was statistically significant resulting in 63.2% mean reduction of tumor growth (see Materials and Methods for calculation of mean reduction of tumor growth). The combination of laminarin-BAM and LPS showed synergistic and strong reduction of tumor growth (mean reduction of tumor growth was 90.2% compared with the control). We observed that 60% of tumors temporarily disappeared or a shrinkage of tumor volume occurred. Decrease of tumor growth was statistically significant compared with the control and with the effect of individual (laminarin-BAM, LPS) components. Regarding survival, its prolongation in the case of a laminarin-BAM/LPS mixture was not statistically significant.

#### Synergy of laminarin-BAM with LPS, various regimes of application

A series of experiments similar to the above mentioned one were performed. Optimization of drug application timing was studied. A mixture of 0.2 mM laminarin-BAM and LPS (0.5 mg/ml) in PBS was used. The results are given in Table 1, highlighting the essential significance of short-term but sufficiently effective therapy.

**Table 1:** Synergy of laminarin-BAM with LPS, various regimes of application.

Application of 0,2 mM laminarin-BAM and LPS (0,5 mg/ml) in 50 μl i.t.	Mean reduction of tumor growth	Statistical significance of survival prolongation	Survival longer than 100 days from the start of therapy
days 0,2,4,6,8,10	83.0%	no	0/5
day 0	64.0%	no	0/5
days 0,1,2	93.9%	no	1/4
day 0 …. 3 doses one hour apart	93.2%	no	1/5
day 1 …. 2 doses one hour apart			
day 2 …. 1 dose			

Groups of 4–5 mice were treated starting the 12^th^ day after tumor transplantation.

#### Use of other mode of laminarin binding to the cell surface

Direct covalent *in vivo* binding of laminarin-SMCC to the cells (with prior reduction of cystines by TCEP) was applied. Laminarin-SMCC (0.2 mM) was administered together with LPS (0.5 mg/ml). This therapy caused stronger reduction of tumor growth than laminarin-BAM/LPS, nevertheless this difference was not statistically significant (data not shown). Reduction (TCEP) and SMCC binding did not influence tumor growth.

#### Control experiments

To demonstrate the necessity of laminarin anchoring to cancer cells, free laminarin was used instead of laminarin-BAM. Laminarin did not reduce tumor growth and its mixture with LPS did not show any signs of additivity or synergy. Tumor growth reducing activity of this mixture corresponded to the activity of LPS alone (data not shown). Anchor alone (lysine-BAM) did not reveal any antitumor activity and its mixture with LPS did not show any signs of additivity or synergy as well.

### The effect of molecules with terminal mannose in cancer therapy

#### Significance of mannose anchoring, the influence of LPS

A 3 mM solution of mannose in PBS did not reduce tumor growth when applied every second day, six injections altogether. Addition of LPS (0.5 mg/ml) did not cause any additivity or synergy, the mixture reduced tumor growth even less than LPS alone. Tumor cells are significantly negatively charged, so we studied their interaction with positively charged mannose-K_10_, containing ten lysine residues chain. Mannose-K_10_ at 3 mM concentration did not influence tumor growth and addition of LPS (0.5 mg/ml) did not cause additivity or synergy. A low effect (32.7% mean reduction of tumor growth compared with the control) was noted using 3 mM solution of mannose-(G)_5)_-(K)_12)_ in PBS, i.e. compound with 5 glycine residue spacer between the ligand and anchoring part of the molecule. This reduction was statistically significant (compared with the control) only on day 6 of therapy (data not shown).

Addition of a lipophilic anchor (mannose-(G)_5_-(K)_10)_-STE) led to a further reduction in tumor growth. A solution of this compound in PBS (3 mM) caused a statistically significant reduction of tumor growth (Figure 1B). Mean reduction of tumor growth was 75,6%. Addition of LPS (0.5 mg/ml) did not cause any additivity or synergy, conversely, mean reduction of tumor growth dropped to 71.2%. The effect of LPS alone remained the strongest. Mice were killed 14 days after the beginning of therapy. The solution of mannose-(G)_5_-(K)_10_-STE fully suppressed appearance of metastases. Incidence and intensity of metastases are summarised in Table 2.

**Table 2:** Influence of intratumoral application of mannose-(G)_5_-(K)10-STE, LPS and combination thereof on incidence of metastases of melanoma B16-F10.

Therapy	Incidence of metastases (%)	Intensity of metastases (mean count of metastases in metastases bearing mice)
3 mM mannose-G_(5)_-K_(10)_-STE	0.0	0
3 mM mannose-G_(5)_-K_(10)_-STE + LPS (0.5 mg/ml)	16.7	2
LPS (0.5 mg/ml PBS)	16.7	5
Control – PBS	50.0	4.3

Groups of 6 mice were examined for the presence of metastases 14 days after beginning of the therapy.

#### The effect of anchored mannan (mannan-BAM) on tumor growth and its synergy with LPS

Mice were treated with mannan-BAM, LPS and a mixture of the two(Figure 1C). Mannan-BAM caused a weak (50.5%), but statistically significant reduction of tumor growth. The effect of LPS was slightly higher (mean reduction of tumor growth was 63.2%). A combination of both compounds caused a strong synergistic reduction of tumor growth (88.6% compared with the control) and tumors temporally disappeared in 80% of mice. The decrease of tumor growth caused by mannan-BAM/LPS mixture was initially statistically significant compared with both control and both individual components of the mixture, later only with the control. Prolongation of mouse survival, caused by the treatment with the mixture of mannan-BAM/LPS, was not statistically significant.

#### Synergy of mannan-BAM with LPS, various regimes of application

**Figure 2:**
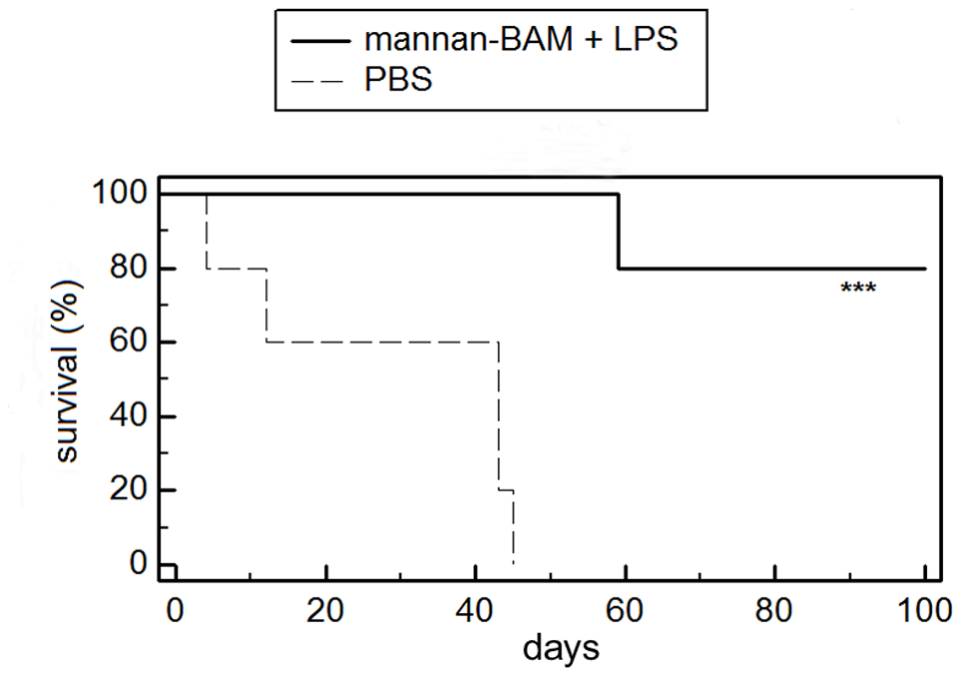
The effect of mannan-BAM/LPS mixture (pulse application) on mouse survival. Mixture of 0.2-BAM and LPS (0,5 mg/ml) in PBS was applied i.t. in pulse regime (days 0,1,2. 8,9,10.16,17,18.24,25,26). Both treated and control group contained 5 mice each. ***indicates P≤0.005 compared to control.

An optimum regime was best achieved by pulse intratumoral application of 50 μl of 0.2 mM mannan-BAM and LPS (0,5 mg/ml) mixture on days 0, 1, 2. 8, 9, 10.16, 17, 18.24, 25, 26. This regime caused not only significant reduction of tumor growth (94,7%) but also statistically significant prolongation of survival (P≤0.005), see Figure 2. An 80% survival rate for 100 days was observed.

#### Use of other mode of mannan binding to the cell surface

Direct covalent *in vivo* binding of 0.2 mM mannan-SMCC to cells (primarily reduced by TCEP) was tested. Mannan-SMCC was administered together with LPS (0.5 mg/ml). As shown in Table 3, high reduction of tumor growth and high ratio of mice with temporary vanishing tumors were observed. The use of four therapeutic pulses of mannan-SMCC/LPS mixture caused almost statistically significant prolongation of survival. Only in this case, survival longer than 100 days was observed.

**Table 3:** Melanoma therapy based on the use of mannan covalently bound to tumor cell surface, synergy with LPS.

Therapy based on TCEP reduction followed by treatment with 0.2mM mannan-SMCC and LPS (0.5 mg/ml). Days of application	Mean reduction of tumor growth	Statistical significance of survival prolongation	Number of mice where tumors disappeared temporarily	Survival longer than 100 days from the start of therapy
0,1,2	92.6%	no	3/5	0/5
0,1,2,8,9,10	98.3%	no	5/5	0/5
0,1,2,8,9,10,16,17,18,24,25,26	97.6%	P = 0.051	5/5	1/5
0,2,4,6,8,10	98.3%	no	4/5	0/5

Groups of 5 mice were treated starting the 12^th^ day after tumor transplantation.

#### Control experiments

As mentioned previously, free mannose did not reduce tumor growth. Its mixture with LPS also did not show any signs of additivity or synergy, all tumor reducing activity of the mixture corresponded to the effect of LPS alone. The same results were obtained with free mannan (data not shown). Testing of new anchoring principles (electrostatic interactions, cell reduction by TCEP and SMCC binding) did not reveal any antitumor activity and combination with LPS did not show any signs of additivity or synergy as well. BAM anchoring did not reveal any anticancer activity as was already described. Regarding (G)_5_-(K)_10_-STE, as described below, no anticancer activity was connected with this type of anchoring as well.

### The effect of formylpeptide receptor agonists in cancer therapy

#### Significance of anchoring of formylpeptide receptor agonists

**The influence of LPS.** In the first experiment, agonists of formylpeptide receptors were attached to the tumor cell's surface on the basis of charge interaction as already mentioned above. f-MLF-(K)_12_ was used as an agonist. Even at 3 mM concentration, it did not reduce growth of the melanoma. The agonist effect was enhanced by using a spacer (5 glycine residue chain), which enables higher flexibility of the terminal f-MLF group. The structure of the above mentioned compound was f-MLF-(G)_5_-(K)_12_. As shown in Figure 1D, the f-MLF-(G)_5_-(K)_12_ solution caused weak, but nevertheless statistically significant reduction of tumor growth (mean reduction of tumor growth was 59.7%), which was significantly enhanced by addition of LPS to 78.3% mean reduction of tumor growth. The f-MLF-(G)_5_-(K)_12_/LPS interaction should be considered slightly additive, as their mixture showed only a slightly higher effect than the more effective component of the mixture.

The molecule of formylpeptide agonist was further modified. Charge interactions were coupled with anchoring of aliphatic chain in lipid layer of cytoplasmic membrane. The structure of this compound was f-MLF-(G)_5_-(K)_10_-STE. As demonstrated in Figure 1E, f-MLF-(G)_5_-(K)_10_-STE acts comparably (55.0% mean reduction of tumor growth) as the compound without stearic acid, used in previous experiment (59.7%). Combination of f-MLF-(G)_5_-(K)_10_-STE with LPS led to a strong synergistic effect, showing marked reduction of tumor growth (98.7%). This reduction was statistically significant in comparison with both components of the mixture. Tumors in five of six mice (83.3%) temporarily disappeared. The increase of survival time in this group was statistically significant (P≤0.05).

#### The use of other modes of binding of f-MLF to the cell surface

A series of experiments revealed that anchored 0.5 mM f-MLF motive in mixture with LPS (0.5 mg/ml) is sufficient for strong reduction of tumor growth. Using these concentrations and various ways of anchoring and timing we performed experiments with the goal to find the best conditions for the strongest antitumor effect.

Results are summarised in Table 4. Experiments confirmed the essential significance of short but sufficiently effective initial therapy, where the mixture of 0.5 mM f-MLFKK-DOPE and LPS (0.5 mg/ml) proved to be the best. 60% of mice treated this way survived 100 days, living further without any pathological symptoms.

**Table 4:** Melanoma therapy using f-MLF bound by various ways to tumor cell surface; synergy with LPS.

Therapy	Mean reduction of tumor growth	Statistical significance of survival prolongation	Number of mice where tumors disappeared temporarily	Survival longer than 100 days from the start of therapy
0.5 mM f-MLFKK-BAM + LPS (0.5	73.9%	no	3/5	0/5
mg/ml), application:				
day 0 …. 3 doses one hour apart				
day 1 …. 2 doses one hour apart				
day 2 …. 1 dose				
0.5 mM f-MLFKK-DOPE + LPS	79.3%	no	3/5	3/5
(0.5 mg/ml), application:				
day 0 …. 3 doses one hour apart				
day 1 …. 2 doses one hour apart				
day 2 …. 1 dose				
0.5 mM f-MLFKK-SMCC + LPS	74.7%	no	2/6	0/6
(0,5 mg/ml), (prereduction+),				
application on days 0,1,2				

Groups of 5–6 mice were treated starting the 12^th^ day after tumor transplantation.

reduction of cystins on cancer cells by 50 mM solution of TCEP in PBS.

#### Control experiments

Free 3 mM f-MLF did not show any reduction of tumor growth and reduction activity of its mixture with LPS corresponded to the activity of LPS alone. Data not shown. Anchors (DOPE as lysine-DOPE, (G)_5_-(K)_10_-STE as immunologically inert MLF-(G)_5_-(K)_10_-STE) did not show any antitumor activity and combinations with LPS did not show any signs of additivity or synergy.

### Analysis of the cell infiltrate in tumors using flow cytometry. Cytokine assays

Three experiments of the same design were performed with three different phagocytic receptor ligands: laminarin-BAM, mannan-BAM and f-MLFKK-BAM alone or in combination with LPS.

**Figure 3:**
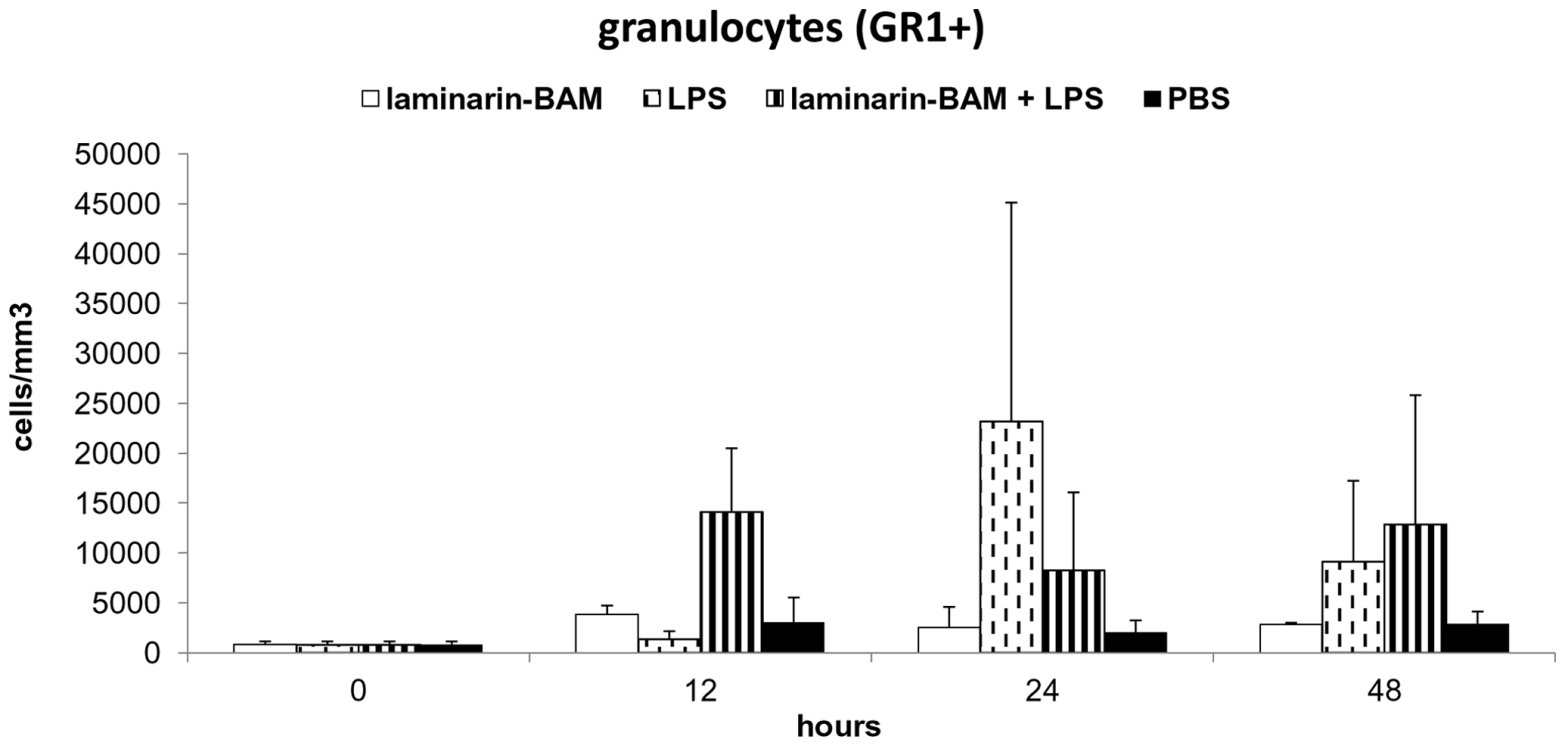
Analysis of cell infiltrate in the tumor during therapy based on the use of laminarin-BAM, LPS and their mixture. Granulocyte detection. Groups of 9 mice received a single dose of 0.2-BAM in PBS, LPS (0.5 mg/ml PBS), mixture of 0.2 mM laminarin-BAM and LPS (0.5 mg/ml) in PBS, and PBS alone in 50 μl i.t. 3 mice from each group were killed in 12, 24 and 48 hours intervals, cells from excised tumors were prepared by enzymatic treatment (Liberase DL and DNase I) and analysed by flow cytometry. For granulocyte detection anti-Mouse Ly-6G (Gr-1) Alexa Fluor 700 was used.

**Figure 4:**
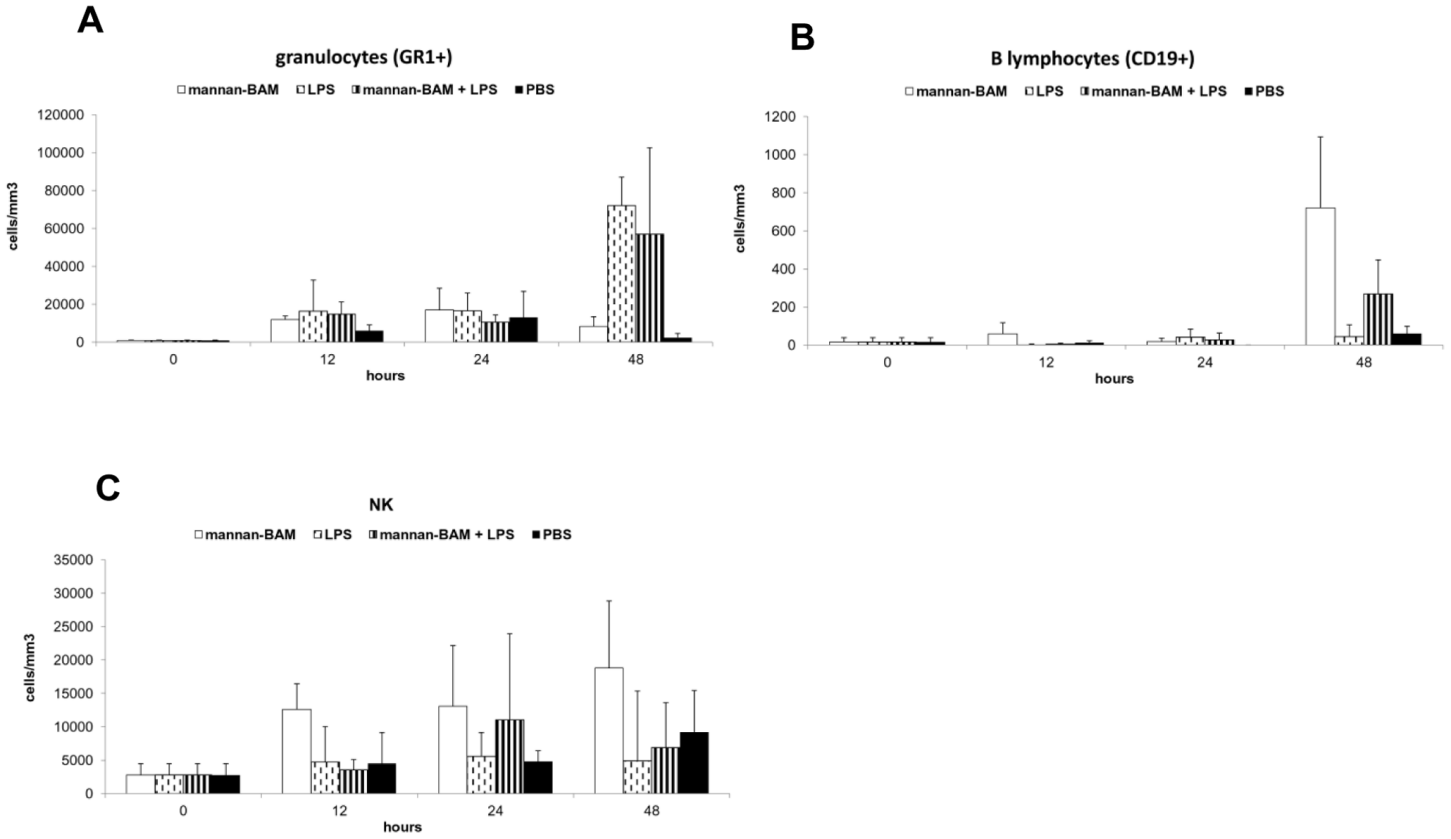
Analysis of cell infiltrate in the tumor during therapy based on the use of mannan-BAM, LPS and their mixture. Groups of 9 mice received a single dose of 0.2-BAM in PBS, LPS (0.5 mg/ml PBS), mixture of 0.2 mM mannan-BAM and LPS (0.5 mg/ml) in PBS, and PBS alone in 50 μl i.t. 3 mice from each group were killed in 12, 24 and 48 hours intervals, cells from excised tumors were prepared by enzymatic treatment (Liberase DL and DNase I) and analysed by flow cytometry. The following labelled antibodies were used: (A) anti-Mouse Ly-6G (Gr-1) Alexa Fluor 700 for granulocyte detection, (B) anti-Mouse CD19 APC for detection of B lymphocytes and (C) anti-Mouse NK1.1 PE for NK cells.

**Figure 5:**
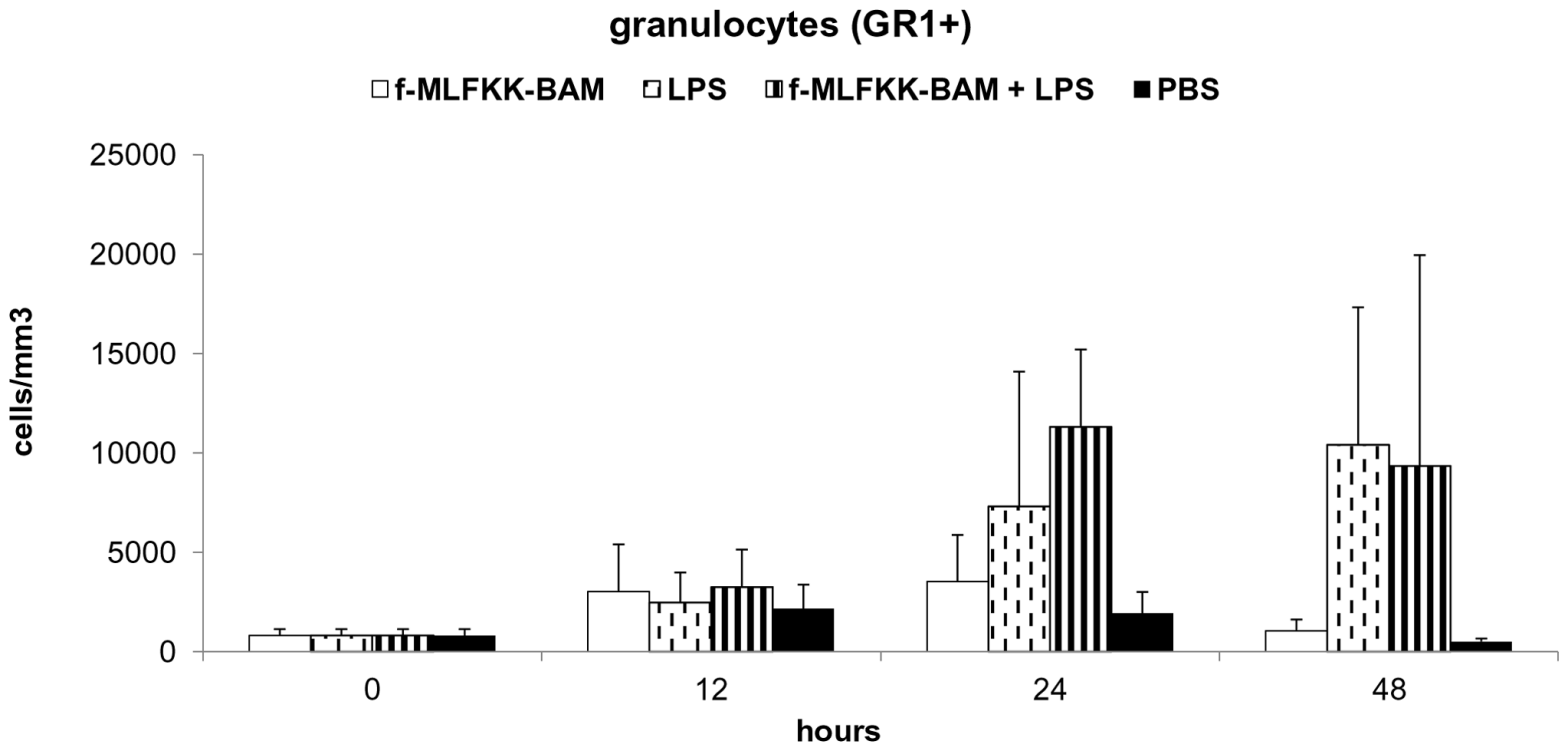
Analysis of cell infiltrate in the tumor during therapy based on the use of f-MLFKK-BAM, LPS and their mixture. Granulocyte detection. Groups of 9 mice received a single dose of 0.5-MLFKK-BAM, LPS (0.5 mg/ml), mixture of 0.5 mM f-MLFKK-BAM and LPS (0.5 mg/ml), and PBS alone in 50 μl i.t. Preparation of cell suspension and granulocyte staining were performed as in Figure 3.

**Figure 6:**
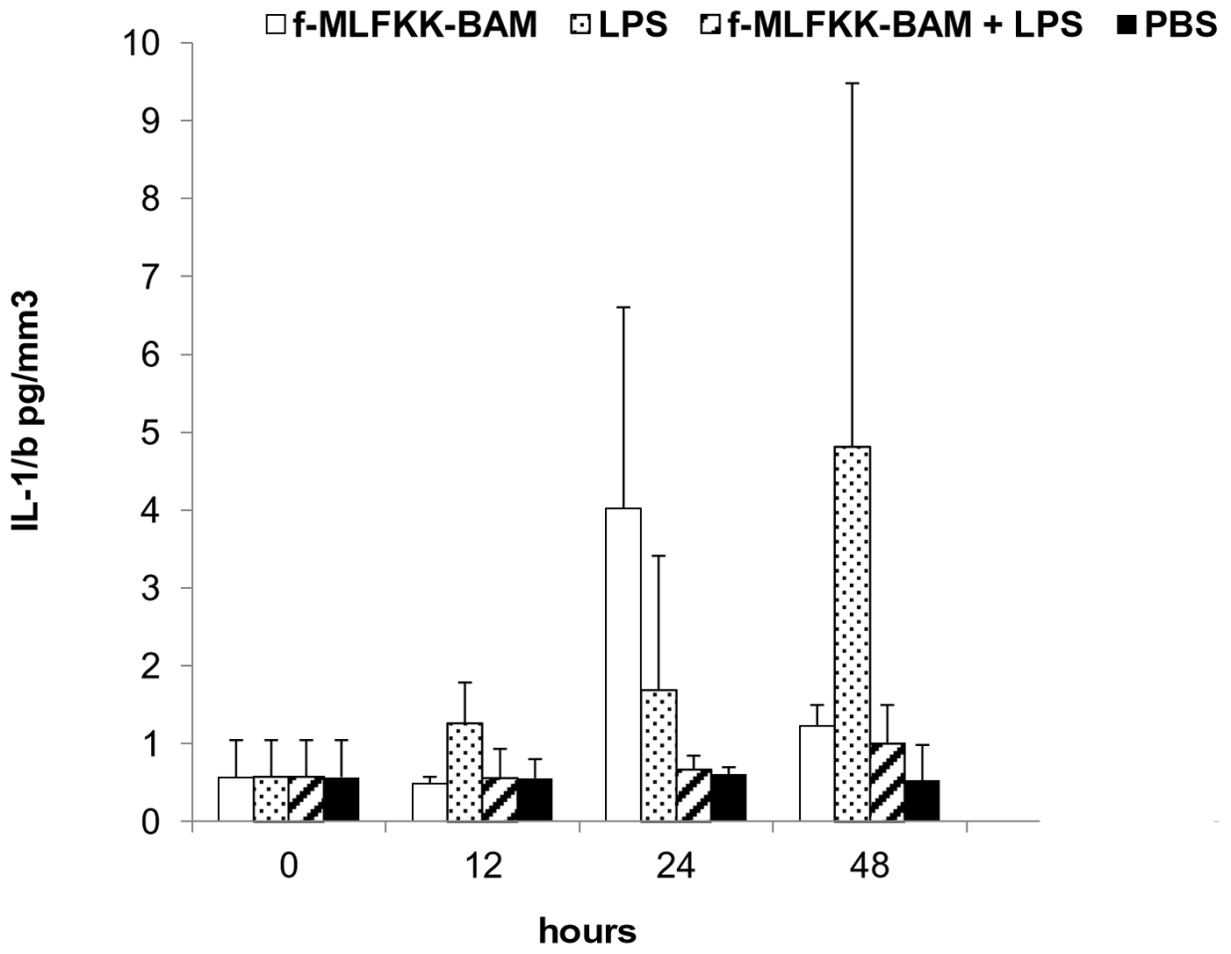
Changes of intratumoral levels of IL-1beta during therapy based on the use of f-MLFKK-BAM, LPS and their mixture. Groups of 9 mice received a single dose of 0.5-MLFKK-BAM, LPS (0.5 mg/ml), mixture of 0.5 mM f-MLFKK-BAM and LPS (0.5 mg/ml), and PBS alone in 50 μl i.t. 3 mice from each group were killed in 12, 24 and 48 hours intervals. After preparation of cells from excised tumors, corresponding supernatants were used for IL-1beta determination. IL-1beta levels are expressed as pg of IL-1beta/mm^3^ of tumor tissue.

In all experiments 3 mice from each group (see legend to Figures 3, 4, 5, 6) were killed in 12, 24 and 48 hour intervals (+3 control mice were killed at time 0). Cells for flow cytometry and supernatants for ELISA were prepared and analysed.

#### Therapy based on the use of laminarin-BAM, LPS and their mixture

Changes in granulocyte count (GR1+) only were observed in the monitored period. A significant increase of their count was detected in laminarin-BAM/LPS and LPS groups (Figure 3). The increase of cell count in the laminarin-BAM/LPS group preceded increase in the LPS group (12 hours difference). These changes were reflected in the total leukocyte count (CD45+) (data not shown). The total number of infiltrating cells during 48 hours was comparable in both groups. No changes in monocyte/macrophage (F4/80+), T lymphocyte (CD3+), CD4+, CD8+, NK, B lymphocyte (CD19+) count were observed.

#### Therapy based on the use of mannan-BAM, LPS and their mixture

The increase of granulocyte count was detected again mainly in mannan-BAM/LPS and LPS groups (Figure 4A). The increase was synchronous in both groups and was reflected by the increase of total leukocytes (CD45+) (data not shown). No significant differences between mannan-BAM/LPS and LPS groups were found. The increase of B lymphocytes (CD19+) and NK cells in mannan-BAM group and partially in the group mannan-BAM/LPS were demonstrated (Figure 4B, 4C).

#### Therapy based on the use of f-MLFKK-BAM, LPS and their mixture

The changes observed correspond to the experiment with laminarin-BAM, LPS and their mixture. An increase of granulocyte (GR1+) count in groups f-MLFKK-BAM/LPS and LPS was observed (Figure 5). The increase of cell count in the group f-MLFKK-BAM/LPS preceded that in the group LPS (24 hours difference). The total number of tumor infiltrating cells during 48 hours of experiment was comparable in both groups. Simultaneous presence of agonists of both signalling and phagocytic receptors led to early culmination of granulocyte infiltration only. No changes in monocyte/macrophage (F4/80+), T lymphocyte (CD3+), CD4+, CD8+, NK, B lymphocyte (CD19+) count were observed.

In all three above mentioned experiments the levels of IL-1beta, TNF-alpha, IL-6, and IL-8 were determined. No signs of synergy between LPS and phagocytic ligands causing increased cytokine levels were observed. Ligands alone and LPS alone caused an increase of all cytokines, which corresponds to the onset of inflammatory processes. Levels of typical proinflammatory cytokine IL-1beta are shown in Figure 6.

### Histology

**Figure 7:**
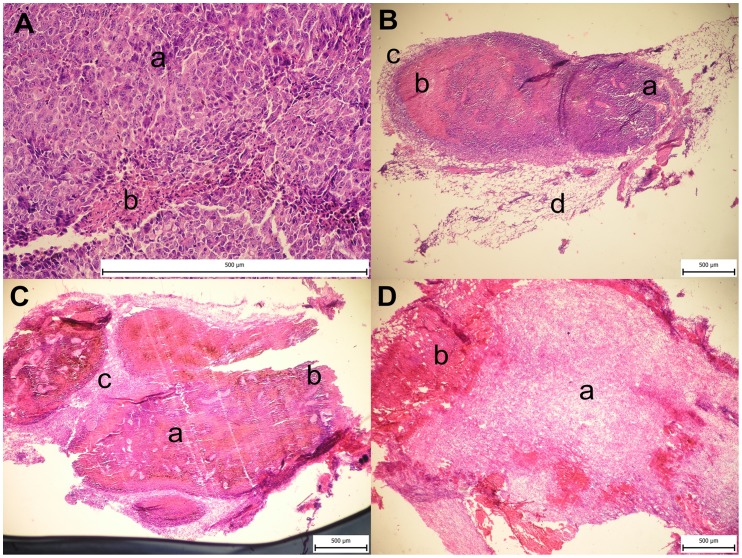
Histology. Melanoma bearing mice were injected i.t. with 50 μl of BAM derivatives of agonists (0.2 mM laminarin-BAM, 0.2 mM mannan-BAM, 0.5 mM f-MLFKK-BAM), their mixtures with LPS (0.5 mg/ml), LPS and PBS alone. Two mice from each group were killed in 24 hours intervals (24, 48, 72 hrs). Excised tumors were fixed with 4% neutral solution of formaldehyde and paraffin blocks were prepared. Sections were stained with hematoxylin/eosin. A– PBS alone; B– effect of particular agonists of phagocytic receptors and LPS alone; C, D– synergistic effect of LPS combinations with particular agonists of phagocytic receptors. Aa – melanoblasts, Ab – necrotic focus with slight granulocyte infiltration, Ba – melanoblasts, Bb – necrotic focus with hemorrhage, Bc – granulation tissue, Bd – slacked edematous ligament, Ca – necrotic tissue with hemorrhage, Cb – negligible residue of tumor, Cc – edematous ligament with inflammatory infiltration, Da – slacked edematous ligament with inflammatory infiltration and hemorrhage foci, Db – bleeding necrosis.

Melanoma bearing mice were treated with phagocytic receptor ligands, laminarin-BAM, mannan-BAM and f-MLFKK-BAM alone, or in combination with LPS. Two mice from each group were killed in 24 h intervals (24 h, 48 h, 72 h). Figure 7A shows negligible granulocyte infiltration in the case of PBS application. Application of particular agonists of phagocytic receptors and LPS alone resulted in partial reduction of tumor structures (Figure 7B). The highest reduction was noted for LPS, followed by laminarin-BAM, mannan-BAM and f-MLFKK-BAM. Infiltration constituted by granulocytes (48 h) changed in favour of monocytes/macrophages (72 h). Combinations of LPS with agonists of phagocytic receptors caused a significant reduction of tumor structures (Figure 7C,D).

### *In vitro* analysis of the effect of macrophages activated by a TLR ligand on melanoma cells bearing phagocytic ligands

#### Anchored laminarin-BAM

**Figure 8:**
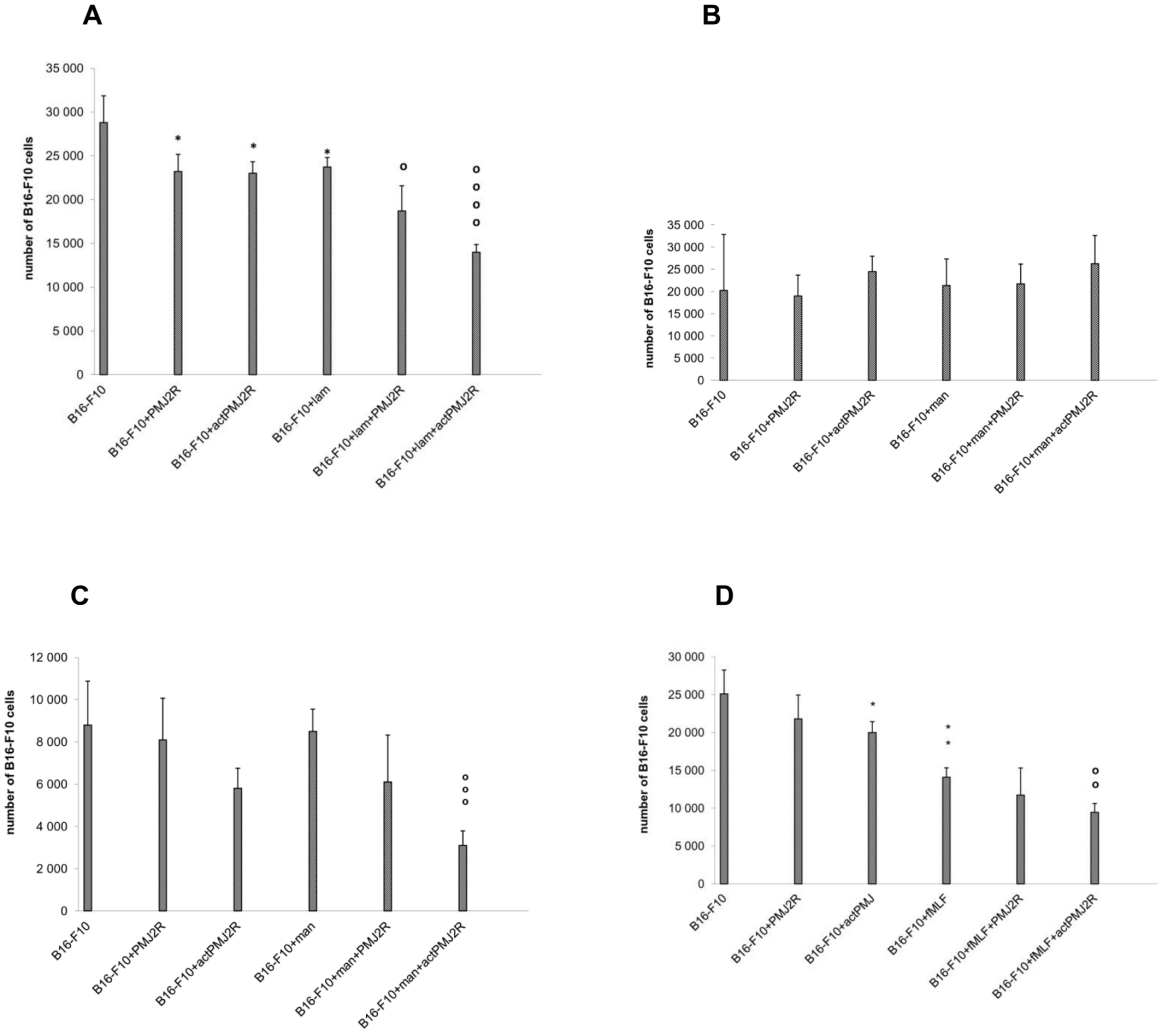
*In vitro* analysis of the effect of macrophages activated by LPS on melanoma cells bearing ligands of phagocytic receptors. Murine B16-F10 melanoma cells grown to confluency in 96 well tissue culture plate were incubated (30 min, 37°C) with solution of phagocytic receptor agonists (0.02 mM laminarin–BAM or 0.02 mM mannan-BAM or 0.05 mM f-MLFKK-BAM in culture medium) and subsequently washed. Cells of murine macrophage cell line PMJ2R were preincubated with LPS (1 μg/ml) for 2 hours at 37°C, washed, and added to B16-F10 in the ratio 5:1. This mixture was incubated for 4 hours at 37°C. After incubation, PMJ2R and dead cells were carefully washed off. Living B16-F10 melanoma cells were released by trypsinisation and calculated. (A) laminarin-BAM, (B) mannan-BAM, (C) mannan-BAM, cells cultured in medium with non-inactivated fetal calf serum, (D) f-MLFKK-BAM. *P≤0.05, **P ≤ 0.005 compared to B16-F10 **o**P≤0.05, **oo**P≤0.005, **ooo**P≤0.0005, **oooo**P≤0.00005 compared to B16-F10+ ligand. The experiment was repeated twice with similar results.

As shown in Figure 8A, the effect of resting or LPS-activated PMJ2R macrophages on melanoma cells was similar and low. Anchoring of the phagocytic ligand on melanoma cells enhanced the cytotoxic effect of intact PMJ2R macrophages only slightly. A statistically significant effect was observed when LPS activated PMJ2R macrophages reduced number of laminarin-BAM bearing melanoma cells by 41%.

#### Anchored mannan-BAM

Neither resting nor LPS activated PMJ2R macrophages caused any effect on melanoma B16-F10 or mannan-BAM bearing melanoma B16-F10 cells (Figure 8B).

#### Anchored mannan-BAM. Medium with native serum

This experiment was performed as the previous one, but with one modification: foetal calf serum was not heat inactivated; hence complement activity was preserved. Resting PMJ2R cells reduced the number of B16-F10 by 8%. LPS activated macrophages caused 34% statistically not significant reduction of B16-F10 cells. The effect of mannan-BAM binding on tumor cell surface was negligible (3% reduction). Resting PMJ2R macrophages reduced the number of ligand-labelled melanoma cells by 28%. LPS activated PMJ2R macrophages reduced the number of mannan-BAM bearing melanoma cells highly significantly (64% reduction) (Figure 8C). The last two experiments proved the role of complement in killing of mannan-BAM bearing melanoma cells.

#### Anchored f-MLFKK-BAM

As shown in Figure 8D, resting PMJ2R macrophages showed a statistically not significant reduction of the number of B16-F10 cells (13%). LPS activated macrophages significantly reduced the number of B16-F10 cells (20% reduction). Anchoring of f-MLFKK-BAM on B16-F10 surface caused significant 44% decrease of B16-F10 cell number. Resting PMJ2R reduced the number of f-MLFKK-BAM bearing melanoma cells (B16-F10+ f-MLF) by 17%. LPS activated PMJ2R reduced the number of f-MLFKK-BAM bearing melanoma cells by 33% (statistically significant).

### Interaction of macrophages with melanoma cells labelled with phagocytic ligands. Formation of clusters

**Figure 9:**
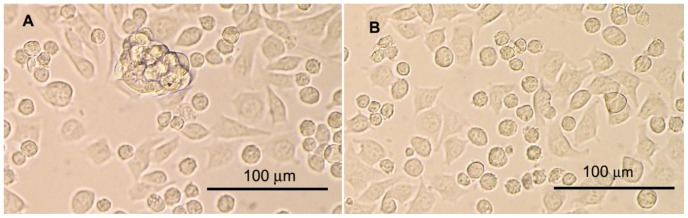
Interaction of macrophages with melanoma cells labelled with phagocytic ligands. Formation of clusters. To the mixture of melanoma B16-F10 cells and macrophage cell line PMJ2R laminarin-BAM (A) or free laminarin (B) were added (0.05 mM final concentrations). Photos were taken after 1 hour incubation at 37°C. The experiment was repeated four times with similar results.

The influence of laminarin, mannan, and f-MLF (free and bound) on interaction of PMJ2R macrophages with melanoma B16-F10 was studied. Formation of macrophage/melanoma clusters was observed when laminarin-SMCC was covalently bound on melanoma cells. In case of f-MLF, optimal conditions for cluster formation were achieved, when f-MLFKK-BAM was added directly to the mixture of both cells (0.05 mM final concentration), see Figure 9A. Free f-MLF did not show any effect (Figure 9B). Both laminarin-SMCC and f-MLFKK-BAM dependent clusters were composed of PMJ2R and melanoma cells, as proved by immunofluorescence using anti-CD11b-FITC conjugate for PMJ2R staining (all nuclei were stained by DAPI). Mannan-dependent formation of clusters was never observed.

### Macrophage activation by laminarin anchored (laminarin-BAM) or covalently bound (laminarin–SMCC) to tumor cells. Cell signalling

**Figure 10:**
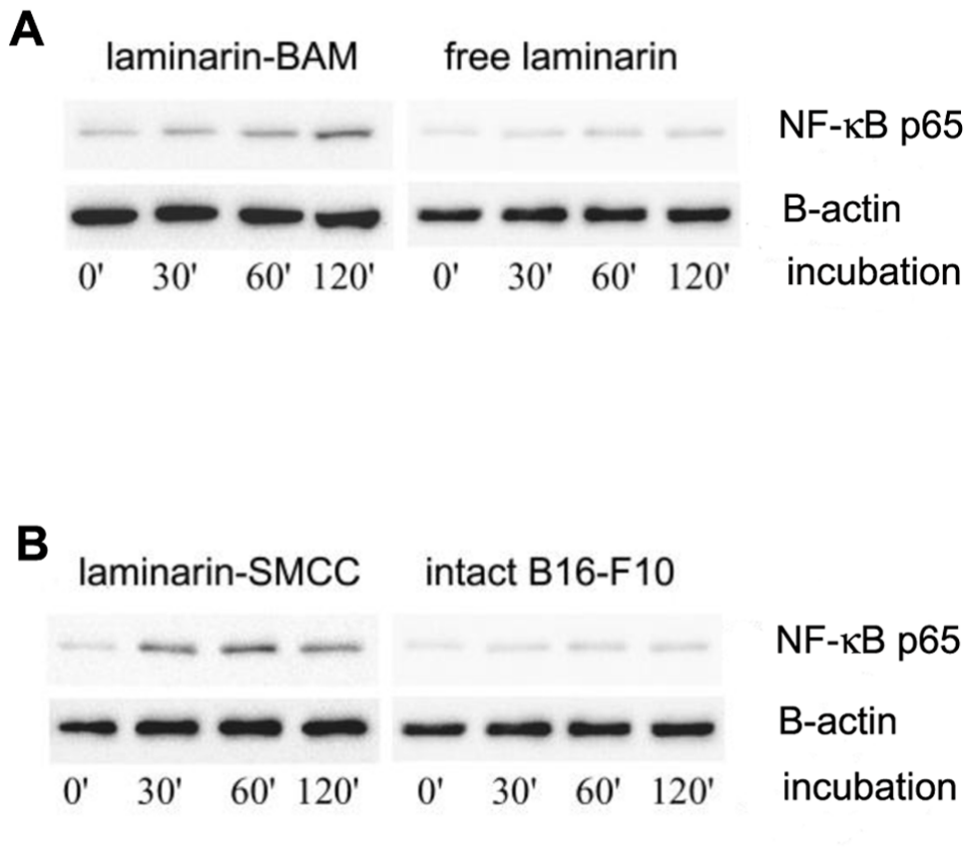
Macrophage activation by laminarin anchored (laminarin-BAM) or covalently bound (laminarin–SMCC) to tumor cells. B16-F10 cells were cultured with PMJ2R cells for indicated time in the presence of 0.05 mM laminarin-BAM. Free laminarin at the same concentration was used as a control. NF-κB kinase activation was assessed by immunoblotting with antibody specifically recognizing only phosphorylated form. β-actin is shown as a loading control. Two independent experiments were performed. Representative blots are shown (A). In further experiment laminarin-SMCC was covalently bound to B16-F10 cells prior seeding with PMJ2R cells. Intact B16-F10 melanoma cells were used as a control. NF-κB kinase activation was assessed as previously described. Resulting blots are shown (B).

To confirm that laminarin anchored to tumor cells activates macrophage cells we measured the phosphorylation of kinase NF-κB p65 (Ser536), a downstream signalling molecule of Dectin-1/SYK signalling pathway [21]. The phosphorylation of NF-κB p65 was determined in coculture of tumor cells and PMJ2R in the presence of laminarin-BAM (0.05 mM final concentration) at indicated times after seeding. Free laminarin at the same concentration was used as a control. As shown in Figure 10A, phosphorylation/activation of NF-κB p65 raised by increasing time of incubation when laminarin –BAM was present in the coculture. Free laminarin did not activate NF-κB p65. Similarly, an increase of NF-κB p65 activation occurred when laminarin-SMCC was covalently bound to B16-F10 cells prior seeding with PMJ2R cells (Figure 10B).

### Capability of BAM and DOPE to anchor molecules to cell membranes

Anchoring of BAM and DOPE with covalently bound B-phycoerythrine (PE) to melanoma cells was studied. Fluorimetric method of PE-BAM, PE-DOPE and PE determination was optimised. The amount of bound compounds was calculated from the decrease of fluorescence of their solutions after incubation with cells. Non-specifically bound molecules (PE background) were subtracted. As shown in Table 5, at higher original concentrations (before binding) both BAM and DOPE offer similar binding capacity. At lower concentrations BAM is more suitable.

**Table 5:** Proof and quantification of PE-BAM and PE-DOPE conjugates anchored to the cell surface.

Compound	Concentration(nmol/l)	Amount of compounds bound to 100,000 melanoma cells (fmol)	Number of molecules specifically bound (anchored) to one melanoma cell
PE-BAM	2.5	104.84	338,211
	1.25	46.36	150,482
	0.625	27.91	68,889
PE-DOPE	2.5	113.80	392,186
	1.25	33.35	72,164
	0.625	18.99	15,151
PE	2.5	48.68	0
	1.25	21.37	0
	0.625	16.47	0

All measurements were done in triplicates.

## Discussion

The use of agonists of Toll-like receptors in cancer treatment has been tested many times. Chicoine et al. [22] achieved significant tumor regression using intratumorally applied LPS. Nevertheless, their model was considerably artificial (subcutaneously implanted mouse glioblastom) and to obtain tumor regression, high and very toxic doses of LPS (200 μl solution of 2–2.5 mg LPS/ml PBS) were used. In our preliminary experiments (data not shown) we found that LPS solution in concentrations 1 mg LPS/ml and higher is directly cytotoxic, hence, the above mentioned study cannot be considered solely immunological. We are using solution of 0.5 mg LPS/ml. This solution did not show any cytotoxicity either for melanoma or normal not transformed cells (primary culture of guinea pig kidney cells) during 24 hour cultivation (*in vitro* experiments, data not shown). Fifty microliters of the mentioned solution was applied intratumorally in our experiments, which corresponds to 25 μg of LPS only. Mariani at al. [23] achieved inhibition of tumor growth by intratumoral applications of LPS using rat glioma RG-2 cells implanted subcutaneously. It was necessary to use high LPS doses (intratumoral application of 50 μl of LPS at concentration as high as 100 mg LPS/ml saline, hence 5 mg of LPS). Reduction of tumor growth based on intratumoral injection of flagelline (TLR5 agonist) was studied by Rhee et al. [24]. The model used was again artificial (human colon carcinoma transplanted to nu-nu mice), hardly comparable with the fast growing aggressive melanoma B16-F10 exploited in our studies. Synthetic analogues of signal receptors used by pharmaceutical industry have not yielded satisfactory results in cancer treatment [25].

The possibility of using phagocytic receptor agonists in cancer therapy was proved in our study.

First it was necessary to demonstrate that compounds used in our *in vivo* experiments activate innate immunity via activation of phagocytic receptors. It was guaranteed not only by the use of specific well described [26], [27] ligands of these receptors. Binding of phagocytic receptor agonists to corresponding receptors has to be strengthened by multiplications of these bonds [28], hence only ligands anchored to the cell surface are active. It corresponds well with our experiments. Moreover, the anticancer activity of all compounds tested was fully dependent on molecular motives interacting with phagocytic receptors; no role of anchoring system was detected. Observed onset of inflammation with characteristic picture (increase of proinflammatory cytokines and infiltration of inflammatory cells) indicated participation of considered mechanisms. All *in vitro* experiments with macrophages also supported our opinion of the role of bound phagocytic ligands (ligands which did not bind to B16-F10 cells were washed out). BAM and DOPE are commercial tools for anchoring molecules to cells; nevertheless, we verified their binding capacity to melanoma cells by direct fluorescence based quantitative measurement *in vitro*.

Nevertheless, the effect of anchored agonists of phagocytic receptors caused only partial reduction of tumor growth, comparable with the effect of agonists of TLR. The combination of soluble agonists of TLR and membrane anchored agonists of phagocytic receptors was proven to be the key step. This combination led to huge synergistic reaction, causing strong reduction of tumor growth. Frequent shrinkage and even vanishing of tumors was observed. The vanishing was often temporary. Statistically significant prolongation of survival and frequently complete recovery of mice were achieved by appropriate therapeutic regimes (mice are so far living for hundreds of days). As all interest was concentrated on long lasting survival, the question of metastases was solved only partially. Nevertheless, it is clear that the therapies used suppress metastases as well. Melanoma is a strongly metastasing tumor, and without this suppression mice would be killed by metastases.

Underhill and Gantner [28] described complex interplay of TLR signalization (soluble ligands) and activation of phagocytic receptors (bound ligands), leading to a coordinated inflammatory response. In the area of tumor therapy, this complexity has never been considered and applied.

As the first agonist of phagocytic receptors, low molecular weight soluble laminarin was chosen. Laminarin belongs to β-glucans, whose antitumor properties have been clearly proved in the last 40 years [29]. High molecular weight β-glucans applied per orally are used most frequently. The mechanism of their effect (stimulation of granulocyte, monocyte and macrophage activity) was described by Chan et al. [30]. Nevertheless, detailed knowledge of the mechanism of action is still missing [29]. We were able to achieve as much as 50% reduction of tumor growth with per orally administered high molecular weight β-glucan (*Sacharomyces cerevisiae*) (data not shown). Intratumoral application of high molecular weight β-glucan did not show any effect, similar to intratumoral application of laminarin. Lack of effect of laminarin was not surprising, and corresponded to the knowledge of low molecular weight β-glucans [31]. Soluble laminarin is used as effective inhibitor of Dectin-1 [32]. This is in contrast with our goal to activate Dectin-1, an important phagocytic receptor (expressed on macrophages, neutrophils and dendritic cells). Therefore we anchored laminarin to cancer cells using BAM. This way, a prerequisite for Dectin-1 binding was created. Melanoma cells represent the predominant cell population in the tumor (proved by flow cytometry, data not shown), therefore phagocytic attack was directed against them. When LPS was added, this attack was dramatically enhanced (strong synergy), regardless of partial inhibition of Dectin-1 expression under the influence of LPS described by Willment et al. [33].

As a second agonist of phagocytic receptors, terminal mannose was studied. Terminal mannose is recognised by mannose receptor (MR) occurring mainly on macrophages [34]. Activation of complement by mannose (mannan) binding lectin (MBL) must also be considered. This results in both cell opsonization on C3b level, and formation of cytotoxic terminal complexes. Down-regulation of MR by LPS [35] must also be taken into account. Nevertheless, good therapeutic results were obtained in our experiments.

F-MLF was studied as the last agonist of phagocytic receptors. It stimulates formylmethionine phagocytic receptors (FPRs). Seven FPRs were described in mice, three in humans [36]. In this case, in contrast with MR, no inhibition caused by LPS was described. On the contrary, LPS supports expression of FPRs genes in murine macrophages and neutrophils [37]. Therefore, all experiments with a combination of LPS and anchored f-MLF resulted in strong reduction of tumor growth.

Humans are 1000–10,000 times more sensitive to LPS than mice [38]. The reason is the absence of, so far not well defined, serum proteins, which are able to block the majority of LPS in rodents. In the case of using LPS in combination with phagocytic receptors in human therapy, it will be necessary to work with very low, safe concentrations of LPS. Another way is to replace LPS with other agonists of TLR, like LTA or flagellin.

Using a combination of TLR and phagocytic receptor ligands we achieved 80% (mannan-BAM + LPS) and 60% (f-MLFKK-DOPE + LPS) long lasting survival (more than 100 days). To achieve long lasting survival, it is necessary to use well anchored agonists of phagocytic receptors, in an appropriate combination with agonists of TLR and the right timing of therapy. Pulse regime and intensification of therapy at its beginning proved to be very effective.

In the case of combination of LPS with f-MLF-(G)_5_-(K)_10_-STE or with mannan SMCC (some regimes), we achieved very strong reduction of tumor growth (more than 98%) and temporary disappearance of the majority of tumors. Nevertheless, the treatment did not result in long lasting survival and complete recovery of mice. In case of f-MLF-(G)_5_-(K)_10_-STE we suppose that the reason for this could be splitting of oligolysine chain by trypsin and trypsin–like proteases of tumor origin, so the agonist had limited lifespan. In the case of mannan SMCC, we suppose that molecules interacting on charge principle or on the basis of hydrophobic anchoring (BAM, DOPE) can be released from damaged cells and attack new cells again. Molecules bound covalently act well but not repeatedly. This hypothesis has to be proven.

Our *in vitro* experiments showed that agonists of phagocytic receptors anchored to tumor cell surface enhance cytotoxic effect of resting phagocytes and especially of phagocytes activated by a TLR ligand. Flow cytometry analysis of cell infiltrate performed in *in vivo* experiments revealed that the presence of a mixture of TLR and phagocytic receptor agonists results in faster commencement of inflammatory infiltration. The overall magnitude of infiltration was the same as when individual agonists were applied. This phenomenon however was not observed in case of mannan (discussed below).

On the basis of these analyses it is not possible to clarify to a full extent the huge antitumor effect of mixtures of TLR and phagocytic ligands observed in *in vivo* experiments. We suppose that this substantial synergy between agonists of phagocytic and Toll-like receptors is based on two events. The TLR ligand induces early and massive inflammatory infiltration of tumors. The effect of this cell infiltrate is directed towards tumor cells, bearing agonists of phagocytic receptors on their surface. As shown by histology, this results in effective killing of tumor cells. The overall antitumor effect could be strengthened by interplay of TLR and phagocytic receptors [28].

Activation of TLR and phagocytic receptors did not always result in synergy. Mannose linked with short peptide and anchored by hydrophobic chain of stearic acid markedly reduced tumor growth, however its administration with LPS was counter-productive. Mannose bound this way probably served as a suitable agonist of the mannose receptor, which is efficiently downregulated by LPS [35]. Mannose as the terminal part of mannan-BAM apparently activated the lectin pathway of complement by means of MBL. This pathway is LPS insensitive. Therefore it was possible to achieve strong synergy between LPS and mannan-BAM. LPS apparently caused massive infiltration of the tumor by phagocytic cells. Opsonization of tumor cells by C3b/iC3b complement components created conditions for the attack of phagocytes against tumor targets. Our *in vivo* experiments correspond well to experiments performed *in vitro,* where the effect of mannan-BAM was dependent on functional complement in culture medium.

Flow cytometry analysis of cell infiltrate did not reveal any signs of mannan-BAM/LPS synergy. As previously described, complement activation and opsonization of tumor cells led to antitumor attack, nevertheless this pathway is probably not connected with interplay of TLR and phagocytic receptors, which could influence inflammatory infiltration.

The strength of binding of phagocytic receptor agonists to tumor cells is very important for the effect of these agonists on tumor growth, and especially for effective synergy with LPS. Tumor cells have a negative surface charge, which is caused by occurrence of sialic acid and phospatidylserine [39], [40]. The binding of phagocytic ligands based on charge interaction (positive charged oligolysine chain) seems to be insufficient. Anchoring of agonists based on aliphatic chain of stearic acid (or oleoyl acid as in BAM) proved to be very suitable. Two chains anchoring (DOPE) in pulse regime gave also good results (prolongation of survival). Application of covalently bound agonists of phagocytic receptors (SMCC) resulted in highly significant reduction of tumor volume and even frequent temporary disappearance of tumors. However, the effect on survival was low.

Both *in vivo* and *in vitro* experiments proved killing of tumor cells dependent on binding of phagocytic receptor ligands to tumor cells. To elucidate mechanisms of these processes, we studied first steps of interaction of phagocytes with ligand bearing tumor cells, i.e. clusters formation and cell signalling. Formation of clusters, the first step of interaction of phagocytes with melanoma cells, was observed in case of laminarin and f-MLF. Despite testing various conditions, no clusters were observed in case of bound mannan.

When ligands are bound to the cell surface in sufficient density, then phagocytic receptor- ligand interaction leads to clustering of receptors followed by intracellular signalling [41]. Activation of Dectin-1 by anchored laminarin was chosen for cell signalling experiments as Dectin-1 is the best-characterized non-opsonic phagocytic receptor [41]. Experiments confirmed that only bound ligand can trigger phagocytic receptors.

In summary, we have found novel principles of effective cancer therapy. The therapy is based on the use of anchored agonists of phagocytic receptors especially in combination with stimulation of cell signalling receptors like TLR4. Further, we would like to design agonists of phagocytic receptors which will bind specifically to tumor cells. The replacement of LPS with human-safe agonists of signalling receptors and various routes of therapeutic mixtures administration will be a matter of further research.
